# The Short-Chain Fatty Acid Methoxyacetic Acid Disrupts Endogenous Estrogen Receptor-α–Mediated Signaling

**DOI:** 10.1289/ehp.0900800

**Published:** 2009-06-16

**Authors:** Derek V. Henley, Stephanie Mueller, Kenneth S. Korach

**Affiliations:** 1 Receptor Biology Section, Laboratory of Reproductive and Developmental Toxicology, National Institute of Environmental Health Sciences, National Institutes of Health, Department of Health and Human Services, Research Triangle Park, North Carolina, USA; 2 German Cancer Research Center, Systems Biology of Signal Transduction, Heidelberg, Germany

**Keywords:** estrogen receptor, estrogen, methoxyacetic acid, short-chain fatty acid

## Abstract

**Background:**

Ethylene glycol monomethyl ether (EGME) exposure is associated with impaired reproductive function. The primary metabolite of EGME is methoxyacetic acid (MAA), a short-chain fatty acid that inhibits histone deacetylase activity and alters gene expression.

**Objective:**

Because estrogen signaling is necessary for normal reproductive function and modulates gene expression, the estrogen-signaling pathway is a likely target for MAA; however, little is known about the effects of MAA in this regard.

**Methods:**

We evaluated the mechanistic effects of MAA on estrogen receptor (ER) expression and estrogen signaling using *in vitro* and *in vivo* model systems.

**Results:**

MAA potentiates 17β-estradiol (E_2_) stimulation of an estrogen-responsive reporter plasmid in HeLa cells transiently transfected with either a human ERα or ERβ expression vector containing a cytomegalovirus (CMV) promoter. This result is attributed to increased exogenous ER expression due to MAA-mediated activation of the CMV promoter. In contrast to its effects on exogenous ER, MAA decreases endogenous ERα expression and attenuates E_2_-stimulated endogenous gene expression in both MCF-7 cells and the mouse uterus.

**Conclusions:**

These results illustrate the importance of careful experimental design and analysis when assessing the potential endocrine-disrupting properties of a compound to ensure biological responses are in concordance with *in vitro* analyses. Given the established role of the ER in normal reproductive function, the effects of MAA on the endogenous ER reported here are consistent with the reproductive abnormalities observed after EGME exposure and suggest that these toxicities may be due, at least in part, to attenuation of endogenous ER-mediated signaling.

Methoxyacetic acid (MAA) is the primary metabolite of the industrial solvent ethylene glycol monomethyl ether (EGME), which has been used in a variety of coatings and as a jet fuel additive (Gargas et al. 2000; Miller et al. 1983). Interest in EGME and MAA stems from epidemiologic analyses and laboratory studies that have linked exposure to these compounds with reproductive toxicity. In women, occupational exposure to ethylene glycol ethers has been associated with increased risks of spontaneous abortion and subfertility (Correa et al. 1996), whereas exposed males have decreased sperm counts (Welch et al. 1988). In laboratory studies, EGME has been shown to target the ovarian luteal cell, suppress cyclicity, and inhibit ovulation in female rats (Davis et al. 1997); in male rats, EGME has been reported to reduce testicular size and fertility (Rao 1971). Subsequent studies have shown that many of the untoward reproductive effects observed after EGME exposure can be reproduced by exposure to MAA alone, suggesting that MAA is primarily responsible for the compromised reproductive function associated with EGME exposure (Davis et al. 1997; Foster et al. 1984).

The chemical structure of MAA places it in the short-chain fatty acid family, which includes the antiepileptic drug valproic acid (VPA) and the intestinal bacterial product sodium butyrate (NaB). Interestingly, VPA is also associated with reproductive toxicity, including menstrual abnormalities and polycystic ovaries (Isojarvi et al. 1993; Lofgren et al. 2007; O’Donovan et al. 2002), suggesting the short-chain fatty acids may share similar mechanisms of action that lead to reproductive toxicity. A common feature of MAA, VPA, and NaB is their ability to inhibit histone deacetylases (Boffa et al. 1978; Gottlicher et al. 2001; Jansen et al. 2004; Phiel et al. 2001; Sealy and Chalkley 1978), which suggests that one of their major mechanisms of action may be to alter gene expression via histone hyperacetylation. Microarray analyses have confirmed that MAA and VPA, as well as other histone deacetylase (HDAC) inhibitors, alter gene expression profiles in human cell lines; however, the total number of genes regulated by these compounds is relatively low (Jansen et al. 2004; Reid et al. 2005). In addition to altering gene expression profiles through histone hyperacetylation, several HDAC inhibitors, as well as MAA and other short-chain fatty acids, have been shown through *in vitro* analyses to modulate intracellular signaling pathways such as the MAPK (mitogen-activated protein kinase) pathway, which may contribute to their effects on gene expression and cell viability (Jansen et al. 2004; Jung et al. 2005; Michaelis et al. 2006; Rivero and Adunyah 1996; Witt et al. 2002).

Although the histone deacetylase inhibitory activity of MAA has been characterized, little is known regarding the effects of this compound on estrogen signaling, which is critical to reproductive function in both the male and female (Hewitt et al. 2005). Estrogens use both genomic and nongenomic mechanisms to alter the gene expression patterns and proliferative rates of target tissues and cells (Bjornstrom and Sjoberg 2005). Many of these effects are mediated by estrogen receptors ERα and ERβ, which are differentially expressed transcription factors that bind estrogens and transcriptionally regulate the expression of numerous genes. In light of the role of estrogen signaling in normal reproductive function and gene expression and the reproductive toxicity associated with MAA, we sought to determine what effects MAA might have on estrogen signaling *in vitro* and *in vivo* to gain further insight into the molecular mechanisms of action of MAA.

## Materials and Methods

### Mammalian cell culture

MCF-7 cells (ATCC, Manassas, VA, USA) were cultured in Dulbecco’s modified Eagle medium: Nutrient Mixture F-12 (DMEM/F-12; Invitrogen Corporation, Carlsbad, CA, USA) supplemented with 10% fetal bovine serum (FBS; Atlanta Biologicals, Lawrenceville, GA, USA), penicillin (100 U/mL), and streptomycin (100 μg/mL) and incubated at 37°C in a humidified atmosphere containing 5% CO_2_. HeLa cells (ATCC) were cultured in DMEM (Invitrogen) supplemented with 10% FBS, penicillin (100 U/mL), and streptomycin (100 μg/mL) and incubated at 37°C in a humidified atmosphere containing 5% CO_2_.

### Animals and treatments

All procedures involving animals were approved by the Animal Care and Use Committee of the National Institute of Environmental Health Sciences. All animals were treated humanely and with regard for alleviation of suffering. Ten-week-old ovariectomized C57BL/6 mice (Charles River Laboratories, Raleigh, NC, USA) were housed in plastic cages in a temperature-controlled room (21–22°C) with a 12-hr light/dark cycle. Mice were given NIH 31 mouse chow (Ziegler Bros. Inc., Gardner, PA) and fresh water *ad libitum*. Groups of mice (*n* = 3/group) were treated by intraperitoneal injection with saline, 1 μg/kg 17β-estradiol (E_2_), or 400 mg/kg MAA for 2 hr before necropsy. One additional group was treated with 400 mg/kg MAA 30 min before treatment with 1 μg/kg E_2_ for 2 hr. Animals were killed using CO_2_, and uteri were collected and snap-frozen.

### RNA isolation and real-time PCR analysis

#### Cells

MCF-7 cells were plated into 6-well plates (1 × 10^6^ cells/well) and incubated overnight in DMEM/F-12 medium supplemented with 10% FBS. The next day, the media was aspirated from each well, cells were washed with phosphate-buffered saline (PBS), and fresh media [DMEM/F-12 containing 10% charcoal/dextran-treated FBS (HyClone, Logan, UT, USA)] was added to each well. The cells were incubated overnight and then treated for 24 hr. At the end of the treatment period, the cells were harvested and total RNA was isolated using the RNeasy Mini Kit (Qiagen Incorporated, Valencia, CA, USA) according to the manufacturer’s protocol.

#### Uterine tissue

Frozen uterine tissue was pulverized, and total RNA was isolated using TRIzol reagent (Invitrogen) according to the manufacturer’s protocol.

#### Real-time reverse-transcriptase polymerase chain reaction (RT-PCR)

Synthesis of complementary DNA (cDNA) and analysis of gene-specific cDNA concentrations by real-time PCR were performed as previously described (Deroo et al. 2004). Primers for real-time PCR were designed with Primer Express software, version 2.0 (Applied Biosystems Incorporated, Foster City, CA, USA).

### Western blots

MCF-7 cells were cultured and treated as described above. After treatment, cells were washed with PBS and lysed with M-PER Mammalian Protein Extraction Reagent (Thermo Fisher Scientific, Rockford, IL, USA) containing Halt Protease Inhibitor Cocktail (Thermo Fisher Scientific) according to the manufacturer’s protocol to obtain total protein. Protein concentrations were determined using the BCA Protein Assay Kit (Thermo Fisher Scientific), and equal amounts of protein (20 μg) were separated on NuPAGE Novex 10% Bis-Tris gels (Invitrogen). Proteins were transferred to nitrocellulose membranes and stained using the MemCode Reversible Protein Staining Kit (Thermo Fisher Scientific) to ensure equal protein transfer. Membranes were blocked and incubated with antibodies in Tris-buffered saline containing 5% milk and 0.1% Tween-20. ERα protein levels were evaluated with a rabbit polyclonal antibody (sc-7207; Santa Cruz Biotechnology Inc., Santa Cruz, CA, USA) and a horseradish peroxidase-conjugated anti-rabbit antibody (NA934V; Amersham/GE Healthcare Bio-Sciences Corporation, Piscataway, NJ, USA). ERα protein levels were visualized with ECL Plus (Amersham/GE Healthcare Bio-Sciences) and BIOMAX MR film (Kodak/Sigma-Aldrich Corporation, St. Louis, MO, USA).

### Transfections

HeLa cells were plated at a density of 1 × 10^5^ cells/well into 24-well plates in standard growth medium overnight. The following day the medium was changed to DMEM supplemented with 1% charcoal-dextran stripped FBS (SFBS; Hyclone) and transfected using Fugene 6 (Roche Applied Science, Indianapolis, IN, USA) reagent according to the manufacturer’s protocol. After transfection, the cells were incubated overnight in media supplemented with 10% SFBS. The cells were then treated for 24 hr and harvested and assayed for luciferase and β-galactosidase activities using the Luciferase Assay System (Promega Corporation, Madison, WI, USA) and the β-Galactosidase Enzyme Assay System (Promega).

### Statistical analysis

Data were analyzed for statistical significance using the Mann-Whitney nonparametric test.

## Results

### MAA potentiates exogenous ER-mediated signaling

The reproductive toxicities associated with MAA exposure in both humans and animals are similar to some of the reproductive phenotypes observed in both ERα knockout mice (αERKO) and aromatase knockout mice (ArKO), which suggests that MAA may impart its untoward reproductive effects by compromising estrogen-mediated signaling. Interestingly, despite the parallel phenotypes of MAA exposure and animal models of compromised estrogen signaling, MAA has been shown to enhance exogenous nuclear receptor signaling, including ER signaling (Jansen et al. 2004).

**Figure 1:**
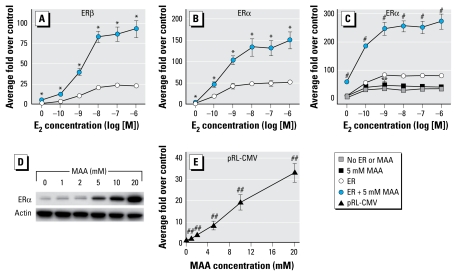
Activation of the CMV promoter by MAA *in vitro*. (*A*) HeLa cells were transfected with a human ERβ expression vector, the estrogen-responsive 3X-ERE-TATA-Luc reporter plasmid, and the CMV-β-gal reporter plasmid, treated for 18 hr with vehicle, increasing concentrations of E_2_, 5 mM MAA, or increasing concentrations of E_2_ plus 5 mM MAA, and assayed for luciferase activity. Data represent the average fold over control (± SE) of duplicate samples from three independent experiments. (*B*) HeLa cells were transfected with a human ERα expression vector, 3X-ERE-TATA-Luc, and CMV-β-gal and treated identically to the cells in (*A*). Data represent the average fold over control (± SE) of duplicate samples from three independent experiments. (*C*) MCF-7 cells were transfected with the 3X-ERE-TATA-Luc reporter plasmid and the CMV-β-gal reporter plasmid with and without the human ERα expression vector. The cells were treated as described for (*A*). Data represent the average fold over control (± SE) of duplicate samples from three independent experiments. (*D*) HeLa cells were transfected with a human ERα expression vector and treated with either vehicle or increasing concentrations of MAA for 18 hr. ERα protein expression was analyzed by Western blot. Data are representative of three independent experiments. (*E*) HeLa cells were transfected with the pRL-CMV reporter plasmid, treated with either vehicle or increasing concentrations of MAA for 18 hr, and assayed for luciferase activity. Data represent the average fold over control (± SE) of duplicate samples from three independent experiments. *p < 0.05 compared with E_2_ treatment. **p < 0.05, and ^#^*p* < 0.01, compared with E_2_ treatment. ^##^*p* < 0.01 compared with vehicle control.

Because these *in vitro* data are incongruent with the reproductive toxicity associated with MAA, we performed similar *in vitro* experiments to assess the effects of MAA on estrogen signaling. We transiently transfected ER-negative HeLa cells with an expression vector for human ERα or ERβ along with an estrogen-inducible 3X-ERE-TATA-Luc firefly luciferase reporter plasmid and a constitutively active cytomegalovirus (CMV)-β-galactosidase reporter plasmid and treated the cells for 24 hr with a solvent control or increasing concentrations of E_2_ in the absence and presence of 5 mM MAA. Consistent with prior observations, our luciferase assay data show that MAA potentiates the activity of E_2_ in HeLa cells transfected with the estrogen-responsive luciferase reporter and either human ERα or ERβ (Figure 1A,B). The ER expression vector is necessary for this response, as identically treated HeLa cells transfected with the reporter in the absence of ERα and ERβ possessed minimal luciferase activity that was unaltered by treatment with E_2_ alone or E_2_ plus MAA (data not shown). In contrast, when ER-positive MCF-7 cells were transfected with only the 3X-ERE-TATA-Luc and CMV-β-galactosidase reporter plasmids and treated identically to the HeLa cells, no potentiation of E_2_-induced luciferase activity was observed with MAA cotreatment (Figure 1C). However, MAA was able to potentiate estrogen-stimulated luciferase activity in MCF-7 cells cotransfected with an expression vector for human ERα (Figure 1C). Interestingly, MAA alone increased luciferase activity relative to vehicle controls in HeLa cells transfected with either ERβ (~ 4.6-fold) or ERα (~ 3-fold) and in MCF-7 cells transfected with ERα (~ 6.5-fold) (Figure 1A–1C). Because the MAA-mediated potentiation of E_2_-stimulated signaling in both cell lines was observed only after transfection with ER expression vectors and because MAA alone was able to increase luciferase activity relative to vehicle controls, we examined the effect of MAA on exogenous ERα expression in HeLa cells. MAA dose-dependently increased the expression of ERα protein in HeLa cells transfected with the human ERα expression vector, which may account for the potentiation of estrogen-induced luciferase activity observed in the transfection experiments with exogenous ER (Figure 1D). We observed this effect in the presence and absence of 10 nM E_2_ (data not shown for E_2_).

The expression vector for both ERα and ERβ is pcDNA3.1, which contains a CMV promoter. To determine if MAA was increasing ER expression by activating the CMV promoter within the expression vector, we transiently transfected HeLa cells with a renilla luciferase plasmid containing a CMV promoter (pRL-CMV) and treated the cells with increasing concentrations of MAA. We observed a concentration-dependent increase in luciferase activity, with maximum activation (~ 30-fold relative to vehicle control) occurring after exposure to 20 mM MAA (Figure 1E). In the same experiments 5 mM MAA induced an ~ 8-fold increase in luciferase activity, indicating that this MAA concentration can significantly activate the CMV promoter. These data suggest that the MAA-mediated potentiation of E_2_-stimulated signaling in these transient transfection experiments is due to an increase in exogenous human ER expression via transactivation of the CMV promoter by MAA. Interestingly, MAA also transactivated the pRL-tk and pRL-SV40 promoters in a dose-dependent fashion, demonstrating 12-fold and 10-fold increases in luciferase activity, respectively, after exposure to 5 mM MAA (data not shown).

### MAA treatment reduces endogenous ER*α* expression

**Figure 2:**
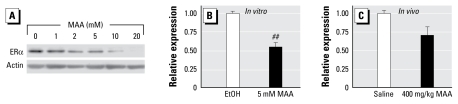
Effect of MAA on endogenous ERα expression *in vitro* and *in vivo*. (*A*) MCF-7 cells were treated for 24 hr with either vehicle [ethanol (EtOH)] or increasing concentrations of MAA, and ERα protein expression was assessed by Western blot. Data are representative of results from three independent experiments. (*B*) MCF-7 cells were treated for 24 hr with either vehicle or 5 mM MAA, and ERα mRNA levels were measured by real-time PCR. Data represent the average fold over control (± SE) obtained from duplicate samples in four independent experiments. (*C*) Uteri were collected from mice treated for 2.5 hr with either saline or 400 mg/kg MAA, and real-time PCR was performed to determine the levels of ERα mRNA in each sample. Data are plotted as fold over control (± SE) and represent the average values obtained from three mice per treatment. ^##^*p* < 0.01 compared with vehicle control.

MAA-induced transactivation of the CMV promoter complicates the interpretation of data obtained from *in vitro* experimental systems incorporating exogenous ER. Therefore, we performed experiments to examine the effect of MAA on the endogenous expression of ERα in MCF-7 cells. MCF-7 cells were treated with increasing concentrations of MAA, and endogenous ERα protein expression was detected by Western blot. We observed a concentration-dependent decrease in endogenous ERα protein expression, with maximal decreases occurring after treatment with 20 mM MAA, the highest concentration tested in these experiments (Figure 2A). To determine if the decrease in ERα protein levels corresponded with decreased steady-state levels of ERα mRNA, MCF-7 cells were treated with 5 mM MAA for 24 hr, and ERα expression was analyzed by real-time PCR. Treatment with 5 mM MAA decreased the expression of ERα mRNA by ~ 50% relative to vehicle controls (Figure 2B), indicating that the decreased protein expression is due, at least in part, to diminished levels of ERα mRNA.

Further experiments were performed in mice to determine if this effect was observed *in vivo*. Ovariectomized C57/BL6 mice were treated for 2.5 hr with either saline or 400 mg/kg MAA, and uteri were collected for measurement of steady-state levels of ERα mRNA by real-time PCR. The dose of MAA used in these experiments was based on a previous report showing that this dose affects nuclear receptor signaling in the mouse uterus (Jansen et al. 2004). MAA decreased ERα expression in the mouse uterus by ~ 30% relative to controls at 2.5 hr (Figure 2C). Although this decrease was not statistically significant, the trend observed in these experiments indicates that MAA has similar effects on endogenous ERα expression *in vitro* and *in vivo*.

### MAA treatment disrupts estrogen-mediated gene expression

**Figure 3:**
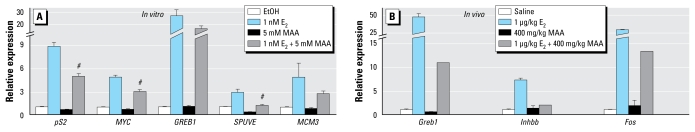
Effect of MAA on estrogen-mediated endogenous gene expression *in vitro* and *in vivo*. (*A*) MCF-7 cells were pretreated with either vehicle [ethanol (EtOH)] or 5 mM MAA for 2 hr and then treated for 18 hr with either vehicle or 1 nM E_2_. The expression of endogenous estrogen-responsive genes was analyzed by real-time PCR. Data represent the average fold over control (± SE) of duplicate samples from at least three independent experiments. (*B*) Mice were pretreated for 30 min with either saline or 400 mg/kg MAA and then treated with either vehicle or 1 μg/kg E_2_ for 2 hr. Uteri were collected and estrogen-responsive gene expression was analyzed by real-time PCR. Data represent the average fold over control (± SE) obtained from three mice per treatment. ^#^*p* < 0.01 compared with 1 nM E_2_ alone.

We performed further experiments to determine if decreased ERα expression after MAA treatment resulted in disrupted ERα-mediated signaling. Toward this end, we treated MCF-7 cells with either 1 nM E_2_, 5 mM MAA, or 1 nM E_2_ plus 5 mM MAA for 24 hr, and evaluated estrogen-regulated gene expression by real-time PCR. As shown in Figure 3A, the expression of *pS2*, *MYC*, *GREB1*, *SPUVE*, and *MCM3* was potentiated by E_2_ treatment; however, pretreatment with 5 mM MAA attenuated the estrogen-induced responses. Taken together, these data show that MAA attenuates endogenous ER signaling, resulting in disruption of estrogen-modulated endogenous gene expression in MCF-7 cells.

To determine whether MAA has a similar effect on estrogen-modulated gene expression *in vivo*, ovariectomized mice were treated for 2 hr with 1 μg/kg E_2_, 400 mg/kg MAA, or 1 μg/kg E_2_ plus 400 mg/kg MAA, and uteri were collected for analysis of gene expression by real-time PCR. The MAA dose used in these experiments is based on a previously published report showing that this dose affects nuclear receptor signaling in the mouse uterus (Jansen et al. 2004). The mRNA levels of the estrogen- inducible genes Greb1, Inhbb, and Fos were increased after treatment with E_2_ alone; however, a 30-min pretreatment with MAA reduced the E_2_-mediated stimulation of each gene (Figure 3B). Although statistical significance was not reached in these experiments, the clear trend in MAA-mediated attenuation of E_2_-stimulated mouse uterine gene expression indicates that MAA has similar effects on *in vitro* and *in vivo* estrogen signaling.

## Discussion and Conclusions

EGME exposure is associated with reproductive toxicity in both humans and animals, and the majority of these effects are attributed to MAA, the primary metabolite of EGME. Despite an established role for estrogen signaling in reproductive function, limited information is available regarding the effects of MAA on estrogen action. Therefore, in the present study we examined the effects of MAA on estrogen signaling, as altered estrogen signaling may be responsible for some of the reproductive toxicity associated with MAA. Our results show that MAA exerts antiestrogenic effects *in vitro* and *in vivo* by reducing endogenous ERα expression and attenuating E_2_-mediated gene expression.

Members of the short-chain fatty acid family such as MAA, VPA, and NaB elicit numerous responses in cells and tissues. One such response that has been described for MAA is the inhibition of histone deacetylase activity (Jansen et al. 2004), which appears to be a common mechanism of action for the short-chain fatty acids (Boffa et al. 1978; Gottlicher et al. 2001; Phiel et al. 2001; Sealy and Chalkley 1978). Because HDAC inhibitors exert a variety of effects on cells and tissues, including altered gene expression, cell cycle arrest, and apoptosis, many of the responses elicited by the short-chain fatty acids are likely associated with their histone deacetylase inhibitory activity. Our results show that, in MCF-7 cells, MAA alone was able to decrease the steady-state mRNA levels of *ER*α (1.9-fold decrease) and the estrogen-responsive genes *pS2* (1.6-fold), *MYC* (1.5-fold), and *SPUVE* (2.7-fold) compared with vehicle controls (Figures 2B and 3A), whereas it increased the expression of *CDKN1C* (4.2-fold increase), a gene that was modestly down-regulated by E_2_ in our experiments (data not shown). However, some genes measured in this study, including *GREB1* and *MCM3*, were not altered by treatment with MAA alone (Figure 3A). These results are consistent with those observed for other short-chain fatty acids and other HDAC inhibitors for which the expression levels of only a small number of genes are significantly altered. For example, treatment of MCF-7 cells with either VPA or trichostatin A (TSA) results in ~ 6% and ~ 20% changes, respectively, in the number of genes whose expression is altered greater than 2-fold as determined from microarray analyses (Reid et al. 2005). A comparison of the gene expression profiles after treatment of MCF-7 cells with MAA, VPA, NaB, sub-eroylanilide hydroxamic acid (SAHA), or TSA shows that many of the same genes are similarly affected by each compound, including *ER*α, *pS2*, *SPUVE*, and *CDKN1C* (Figures 2B and 3A) (Reid et al. 2005). This suggests that most of these alterations in gene expression are due to a common mechanism, which is likely inhibition of histone deacetylase activity.

Because ERα plays an obligatory role in many aspects of estrogen-mediated signaling, our observation that MAA reduces endogenous ERα expression *in vitro* and *in vivo* suggests that estrogen-mediated signaling may be compromised. Indeed, our *in vitro* and *in vivo* analyses confirm that MAA inhibits estrogen-mediated effects on gene expression, showing for the first time that MAA antagonizes E_2_-stimulated expression of ERα target genes. The short-chain fatty acids VPA and NaB have also been shown to reduce ERα expression *in vitro* (Reid et al. 2005; Stevens et al. 1984), suggesting that they may disrupt estrogen signaling as well. Toward this end, VPA, in the absence of E_2_, has been shown to decrease the expression of ~ 90% of the genes that are up-regulated by E_2_ treatment in MCF-7 cells (Reid et al. 2005) and to reduce E_2_-stimulated MCF-7 cell proliferation (Olsen et al. 2004). In addition, NaB, in the absence of estrogen, has been reported to inhibit MCF-7 cell proliferation (Abe and Kufe 1984). Furthermore, NaB attenuates E_2_-stimulated expression of the known estrogen target genes progesterone receptor and *pS2* (De los Santos et al. 2007). Similar results have been observed for the HDAC inhibitors TSA and SAHA, suggesting that histone hyperacetylation may be responsible for the antiestrogenic effects of the short-chain fatty acids (De los Santos et al. 2007; Reid et al. 2005). Our *in vivo* data demonstrate that MAA reduces ERα expression in the mouse uterus by ~ 30% compared with controls (Figure 2C) and attenuates E_2_-stimulated gene expression in the uterus (Figure 3B). The modest decrease in ERα expression in these studies suggests that the changes observed in E_2_-stimulated gene expression may not be due solely to decreased ERα expression, but are likely due to additional mechanisms of action for MAA. This effect is not due to MAA acting as a competitive antagonist for ERα, as MAA does not compete with E_2_ for binding to ERα (Jansen et al. 2004). Taken together, these data suggest that the antiestrogenic effects of the short-chain fatty acids are a class effect that may be due to their inherent HDAC inhibitory activities, because MAA, VPA, and NaB have all been shown to reduce endogenous ERα expression and have been characterized as HDAC inhibitors.

Although MAA imparts antiestrogenic effects on endogenous ER signaling, it enhances estrogen-stimulated reporter activity in the presence of exogenous ERα and ERβ in both HeLa and MCF-7 cells. Similar results have been reported for MAA with respect to the exogenous ER as well as other exogenous nuclear receptors (Bagchi et al. 2009; Jansen et al. 2004). We observed these enhanced responses in MCF-7 cells only when cells were cotransfected with ER expression vectors, indicating that the presence of the ER expression vector is necessary for this effect. In contrast to endogenous ERα expression, which is decreased after MAA exposure, exogenous ERα expression is increased after MAA treatment in the presence and absence of E_2_, and this increase correlates with enhanced luciferase activity in our reporter assays. The underlying mechanism of MAA-induced increases in exogenous ER expression is activation of the CMV promoter, which is present in the ER expression vectors used in this study and is frequently used in other expression vectors. The short-chain fatty acids VPA and NaB and the HDAC inhibitor TSA have also been shown to activate the CMV promoter (Dion et al. 1997; Phiel et al. 2001), which suggests that this is another shared feature of the short-chain fatty acid family and some HDAC inhibitors. The disparate results obtained in our experiments comparing the effects of MAA on endogenous and exogenous ER signaling highlight the importance of our observation that MAA activates the CMV promoter, as each set of results would lead to opposite conclusions regarding the effect of MAA on ER signaling. Based on this observation, careful consideration should be given to experimental design when examining the effects of MAA and other short-chain fatty acids on nuclear receptor signaling to avoid errant conclusions based on experimental artifacts associated with CMV-containing expression vectors. This observation extends to expression vectors containing either TK or SV40 promoters, which were also potently transactivated by MAA in our experiments (data not shown). Similar data have been reported for VPA and NaB with respect to the SV40 promoter, again suggesting a class effect for the short-chain fatty acids (Chen et al. 1999; Gorman et al. 1983).

We have demonstrated that MAA reduces endogenous ERα expression and that MAA treatment inhibits estrogen-mediated endogenous gene expression *in vitro* and *in vivo*. Although extrapolation of the MAA doses used in this study to human exposure levels is challenging given the paucity of data that exists regarding EGME and MAA levels in exposed humans, the antiestrogenic effects of MAA we observed are consistent with the reproductive toxicities described for humans exposed to EGME (Correa et al. 1996; Welch et al. 1988; Welsch 2005). ERα-mediated signaling is critical to reproductive function in both males and females, as illustrated by the phenotypes observed with αERKO mice. Male αERKO mice have reduced sperm counts, and both male and female αERKO mice are infertile (Hewitt et al. 2005). Interestingly, these phenotypes are similar to those observed in EGME-exposed men, who have reduced sperm counts, and women, who exhibit decreased fertility (Correa et al. 1996; Welch et al. 1988). In a rat model, chronic EGME exposure suppressed cyclicity and prolonged diestrus, providing further *in vivo* evidence consistent with attenuation of estrogenic responsiveness (Davis et al. 1997). Adverse reproductive effects have also been reported in men and women exposed to the short-chain fatty acid VPA, which possesses antiestrogenic properties similar to those of MAA (Isojarvi et al. 1993; O’Donovan et al. 2002). Taken together, these observations suggest that MAA-mediated attenuation of ER signaling may play a role in the untoward reproductive effects observed in both males and females after EGME exposure.

## Correction

In the manuscript originally published online, the concentration of E_2_ was given as 1 nM in Figure 1 and in the text referring to the figure. Also, the authors incorrectly noted that MAA potentiates E_2_ activity in HeLa cells in a “dose-dependent manner.” These have been corrected here.
